# Food insecurity in urban and rural areas of Brazil during the COVID-19 pandemic

**DOI:** 10.11606/s1518-8787.2025059006453

**Published:** 2025-10-17

**Authors:** Eloah Costa de Sant Anna Ribeiro, Aline Alves Ferreira, Rosana Salles-Costa

**Affiliations:** I Universidade Federal do Rio de Janeiro. Instituto de Nutrição Josué de Castro. Programa de Pós-graduação em Nutrição. Rio de Janeiro, RJ, Brasil; II Universidade Federal do Rio de Janeiro. Instituto de Nutrição Josué de Castro. Departamento de Nutrição Social e Aplicada. Rio de Janeiro, RJ, Brasil

**Keywords:** COVID-19, Food Insecurity, Brazil, Socioeconomic Factors

## Abstract

**OBJECTIVE:**

To analyze the impact of the COVID-19 pandemic on food insecurity (FI) among families living in rural and urban areas of Brazil.

**METHODS:**

Cross-sectional and descriptive study based on the analysis of two nationally representative surveys conducted using probabilistic sampling by clusters in urban and rural areas of Brazil (2020 and 2022). FI was measured using the Brazilian Food Insecurity Scale. The households were classified as food secure, mild FI, or moderate/severe FI. Prevalence and 95% confidence intervals (95%CI) and analyses were performed in Stata 16 considering the respective sample weights (svy). Variations between the two surveys were analyzed by urban and rural area, and associations with gender and race/skin color.

**RESULTS:**

The majority of households were located in urban areas (2020: 85.6% [n = 1,662]; 2022: 85.5% [n = 10,365]) compared to rural areas (2020: 14.5% [n = 518]; 2022: 85.5% [n = 2,382]). with regard to the characteristics of the household reference person, schooling level, being a formal worker and the per capita family income were higher among families from urban areas. Between 2020 and 2022, the proportion of severe levels of FI increased significantly more in households from rural areas. Despite the higher FI in rural areas, a variation of +54% was noted in urban areas, where the prevalence of moderate/severe FI increased from 19.4% (2020) to approximately 30% (2022). There were greater proportions of FI in households headed by men in urban areas (+75.1%) and mixed race/black people (+55.9%), while households headed by white people saw an improvement in FS.

**CONCLUSION:**

The FI increased unequally between the rural and urban areas of Brazil during the COVID-19 pandemic. The results of this study reinforce the need to plan equitable public policies that debate the different vulnerability profiles aggravated by disparities as a way of guaranteeing food and nutritional security in post pandemic in Brazil.

## INTRODUCTION

The COVID-19 pandemic represents a significant threat to food and nutritional security worldwide. According to the Food and Agriculture Organization (FAO) of the United Nations, the number of people facing hunger in 2022 increased to 90 million^1^. In Latin American and Caribbean countries, the effects of economic recession and social inequalities impacted food insecurity (FI), which increased from 5.6% in 2019 to 7.0% in 2021^2^.

In Brazil, despite the fact that the public policies for Food and Nutrition Security (FNS) and the fight against hunger and poverty implemented in the 2000s have contributed to the reduction of FI, in 2016 the country faced a set of political, economic, and social crises that limited spending on social and health policies^3,4^. This period was marked by setbacks in public policies aimed at food and nutrition and by the increase in IF from 22.6 (2013) to 36.6% (2018)^5^.

In the context of these setbacks, the health crisis generated by COVID-19 further aggravated the inequalities that already existed in the country^5,6^. The health crisis has further aggravated the already existing inequalities in Brazil, with a series of violations of regular and permanent access to quality food in sufficient quantities, that is, the health pandemic occurred together with the hunger pandemic and there were heterogeneous consequences in society^6-8^.

Due to the need to monitor FI during the COVID-19 pandemic, researchers from the Brazilian Network for Sovereignty and Food and Nutritional Security (Rede PENSSAN) carried out the Brazilian Food Insecurity Survey (Vigisan)^7,8^. The data showed that, in 2022, the country had 33.1 million hungry people, and more than 60% of households in rural areas experienced FI. Despite the magnitude of FI in rural areas, a larger population suffered from hunger in urban areas (approximately 27 million people)^8^.

In recent decades, rural households in Brazil have presented the lowest levels of social and FI indicators^9^. However, in urban centres, the unbridled and unplanned growth of cities has resulted in a higher cost of living and territories with economically and socially vulnerable populations^10^. Different aspects can impact FI in rural and urban areas, such as the profile of the household head. Scholars have pointed out that rural families with a female household head, as well as self-declared mixed-race/black families in urban areas, are those most impacted by hunger^11,12^.

In view of these challenges addressed, there is a need to understand how the effect of the COVID-19 pandemic on FI in households located in urban and rural areas. Therefore, the present study aimed to analyse the effect of the COVID-19 pandemic on the FI of families living in rural and urban areas of Brazil.

## METHODS

### Type of Study and Sampling

This was a cross-sectional and descriptive study with microdata from the 2020 and 2022 nationally representative surveys of the National Survey on Food Insecurity during the COVID-19 pandemic (VIGISAN) carried out by the Brazilian Research Network on Food Sovereignty and Food and Nutritional Security (Rede PENSSAN)^7,8^ in December 2020 (1^st^ VIGISAN) and November 2021 to April 2022 (2^nd^ VIGISAN).

Both surveys have similar study designs, with a sample base that is representative of the national territory, considering data from Brazilian Census of 2010 to select the sample size. The households were selected from the same census tracts used in the master sample of the Instituto Brasileiro de Geografia e Estatística (IBGE) population surveys. For the selection of households, conglomerate sampling was used in three stages of selection (municipalities, census tracts, households). The 1^st^ Vigisan obtained a probabilistic sample of 2,180 households, with an estimated 95% confidence interval and a maximum margin of error of 2.1 percentage points for the estimates. In the 2^nd^ Vigisan, 12,745 households were sampled with a maximum margin of error for the total sample of 0.9 percentage points. More details on the sampling design can be found in the methodological section of the surveys. In both surveys, the head of the household was considered the individual responsible for decision-making in the household^7,8^.

More details about pretesting, training, and validation of the collection instrument and data entry were published by Rede PENSSAN^7,8^.

### Food Insecurity

Since 2004, FI has been measured by the Brazilian Household Food Insecurity Scale (EBIA), which assesses food security and FI levels in population studies^13^. This scale was adapted and validated for the Brazilian population and has been used in national research since 2004. The Vigisan used the short version of the EBIA, which consists of eight items and is valid for estimating FI for the full 14 items^14^. The choice to use the 8-item version of the EBIA was due to the need for a rapid population survey to reduce the risk of interviewer contamination.

The EBIA classification establishes four mutually exclusive food security categories and three FI levels based on cut-off points:

- food security (regular and permanent access to quality food in sufficient quantity);
- mild FI (concern or uncertainty about food availability);
- moderate FI (quantitative reduction in food intake and/or disturbance of eating patterns);
- severe FI (presence of food deprivation)^14^.

In this study, the moderate FI and severe FI categories were grouped together as a way of analysing the most severe categories in terms of access to adequate food^12,15^.

### Selected Variables

The individuals responsible for households living in rural or urban areas were included in this study. The profiles of the household heads were considered based on the analysis of gender who was assessed using the biological variable sex, adopted from Brazilian population studies. This variable takes the binary form "male" or "female". However, for the intersectional debate, we used the gender debate to understand the complexities and nuances that extend beyond biological categories, considering how behaviours between men and women shape individual and collective experiences and are related as one of the explanatory arguments, while at the same time structuring inequality. Thus, the variable "gender" will be used to lead the discussions^6^.

Regarding race/skin colour, the self-declaration used in Brazilian population studies was applied, which considers five options: white, black, yellow, brown or indigenous^7,8^. Given that the 1^st^ and 2^nd^ Vigisan address samples that do not account for ethnic and racial minorities, it was not possible to represent the indigenous and yellow categories; thus, both were excluded from the analysis (1^st^ Vigisan: 4.5%; 2^nd^ Vigisan: 3.4%). In Brazil, the black population is composed of both black and brown individuals because both categories are of African descent and face similar challenges due to the racism rooted in Brazilian society. Therefore, in this study, the categories "black" and "mixed-race" were consolidated into a single classification, resulting in the race/skin colour variable having two categories, namely, "white" and "mixed-race/black"^16^.

Socio-demographic variables related to households and the head of the household, which are associated with FI, were assessed for each of the surveys. The covariates were selected based on a theoretical review and a systematic review. The variables include: age group (18 to 49 years old, 50 to 64 years and over 65 years of age), education (up to eight years and more than eight years of study), per capita monthly income categorized into minimum wage equivalences (up to ½, from ½ to one and greater than one minimum wage) considering the value during the research period (based on the minimum wage in Brazil in 2020, US$ 204.80 or R$ 1,039, and, in 2022, US$ 238.90 or R$ 1,212), type of occupation at the interview (formal worker, informal worker and considering formal contracts, self-employed, and unemployed) and macroregion (South/Southeast, North, Northeast and Midwest).

### Data Analysis

Means and standard errors (SEs) were estimated for continuous variables using Student's t-test for comparison. For categorical variables, the percentage and 95% confidence intervals (95%CI) were determined, and the χ^2^ test was used to evaluate associations between the study variables and FI. All variables were tested for normality using the Kolmogorov-Smirnov and Shapiro-Wilk tests. The variations in prevalence were evaluated considering the period between the two surveys (2020–2022), given by Equation 1:


(1) $$[\text{CΔ}=(\text{Py}2-\text{Py}1)/\text{Py}1]{\text{g}}^{\prime }$$

Where:

Py2 and Py1: the prevalences in the years 2022 and 2020, respectively^3,4^.

Statistical analysis was performed considering the complex sampling design and 95%CI using Stata software, version 16.1 (StataCorp LP, College Station, United States) with the survey data analysis command (svy prefix) and considering the complex sample design of the study^17^.

### Ethical Aspects

This study used data made available by the PENSSAN Network. Vigisan is part of a broad project to monitor FI in the context of COVID-19, which was approved by the Research Ethics Committee of the Hospital Universitário Clementino Fraga Filho, Universidade Federal do Rio de Janeiro — Certificate of Presentation for Ethical Appreciation (CAEE) 30679914.0.0000.5257^7,8^.

## RESULTS

It was observed that most households were located in the urban area (2020: 85.6% [n = 1,662]; 2022: 85.5% [n = 10,365]) compared to the rural area (2020: 14.5% [n = 518]; 2022: 85.5% [n = 2,382]). In both areas, more than half of the heads of household were male, self-declared mixed color/black and in the age group of > 18 and < 49 years older. The households headed in urban areas had a higher prevalence of schooling level, formal workers and monthly per capita income than households located in rural areas. Comparing the two surveys, there was an increase in the proportion of households headed by people who had informal employment, especially in rural areas (Table 1).

**Table 1:** Socioeconomic and demographic characteristics by urban and rural areas in Brazil. National Survey on Food Insecurity in the Context of the COVID-19 pandemic in Brazil, 2020 and 2022.

Sociodemographic characteristics		Brazil		Urban area		Rural area	
		2020	2022	2020	2022	2020	2022
		% (95%CI)	% (95%CI)	% (95%CI)	% (95%CI)	% (95%CI)	% (95%CI)
**Sex**	Man	51.3 (47.94–54.64)	51.2 (49.94–52.44)	50.3 (46.53–54.07)	50.4 (49.01–51.82)	57.3 (50.33–63.92)	55.8 (53.14–58.38)
	Woman	48.7 (45.36–52.06)	48.8 (47.56–50.06)	49.7 (45.93–53.47)	49.6 (48.18–50.99)	42.7 (36.08–49.67)	44.2 (41.62–46.86)
**Race/skin color**	White	38.3 (34.20–42.49)	36.5 (35.20–37.75)	39.3 (34.89–43.98)	37.6 (36.38–38.84)	31.5 (22.40–42.35)	29.6 (25.90–33.69)
	Mixed Color/Black	61.7 (57.51–65.80)	63.5 (62.25–64.80)	60.7 (56.02–65.11)	62.4 (61.16–63.62)	68.5 (57.65–77.60)	70.3 (66.31–74.10)
**Age (years)**	> 18 and < 49	47.9 (44.72–51.05)	54.7 (53.59–55.80)	47.4 (43.95–50.97)	55.0 (53.76–56.33)	50.5 (43.89–57.05)	52.6 (48.48–56.75)
	≤ 50 and < 64	33.5 (30.76–36.40)	28.2 (27.30–29.18)	33.8 (30.62–37.06)	28.2 (27.13–29.27)	32.1 (26.19–38.56)	28.5 (26.12–30.95)
	≥ 65	18.6 (15.96–21.57)	17.1 (16.19–18.00)	18.8 (15.85–22.13)	16.8 (15.78–17.79)	17.5 (14.58–20.78)	18.9 (16.44–21.62)
**Schooling level (years)**	> 9	50.3 (47.5–53.1)	52.9 (50.83–54.91)	50.9 (46.35–55.48)	56.2 (54.38–57.99)	30.7 (23.86–38.56)	33.3 (30.15–36.70)
	≤ 8	49.7 (49.9–52.5)	47.1 (45.09–49.17)	49.1 (44.52–53.65)	43.8 (42.01–45.62)	69.3 (61.44–76.14)	66.7 (63.30–69.85)
**Monthly per capita income (minimum wage** a)	> 1 MW	23.9 (20.30–27.82)	30.4 (28.96–31.99)	25.1 (21.32–29.33)	32.3 (30.81–33.88)	16.5 (10.05–25.86)	19.4 (15.87–23.58)
	> 1/2 and ≤ 1 MW	32.8 (29.7–36.15)	32.8 (31.97–33.74)	30.8 (24.04–38.51)	32.8 (31.79–33.85)	33.2 (29.71–36.87)	33.1 (29.89–36.45)
	≤ 1/2 MW	43.3 (38.79–47.9)	36.7 (35.13–38.28)	52.7 (39.59–65.47)	34.9 (33.25–36.51)	41.7 (37.20–46.33)	47.5 (42.88–52.12)
**Type of occupation at the interview**	Formal worker	35.9 (31.7–40.4)	37.5 (35.9–39.1)	35.6 (31.1–40.5)	38.5 (36.7–40.3)	38.8 (29.3–49.3)	29.6 (26.2–33.2)
	Informal worker	10.7 (8.2–13.8)	26.5 (24.9–28.1)	10.6 (8.0–13.9)	24.6 (23.1–26.2)	11.8 (5.7–23.0)	40.7 (37.1–44.4)
	Self-employed	40.0 (35.4–44.8)	24.3 (22.9–25.7)	40.0 (35.1–45.2)	25.0 (23.5–26.6)	39.5 (30.2–49.7)	18.8 (15.2–23.0)
	Unemployed	13.4 (10.4–17.0)	11.8 (10.8–12.8)	13.7 (10.6–17.6)	11.9 (10.8–13.0)	9.8 (5.4–17.2)	10.9 (9.2–12.9)
**Number of residents**	≤ 2	40.6 (37.38–43.8)	50.7 (49.01–52.35)	40.6 (37.16–44.22)	50.4 (48.69–52.05)	40.1 (32.99–47.75)	52.5 (48.24–56.70)
	> 2 and ≤ 4	45.2 (42.1–48.26)	38.4 (36.84–39.96)	45.5 (42.18–48.83)	38.7 (37.06–40.40)	43.3 (35.63–51.30)	36.5 (33.39–39.70)
	> 4	14.3 (12.0–16.84)	10.9 (10.26–11.63)	13.9 (11.45–16.71)	10.9 (10.24–11.62)	16.6 (12.41–21.75)	11.0 (9.12–13.28)
**Region of Brazil**	Southeast/South	58.6 (51.2–65.66)	59.5 (57.15–61.81)	62.7 (54.61–70.08)	63.2 (60.69–65.65)	34.5 (20.20–52.31)	37.7 (30.38–45.64)
	North	7.5 (5.48–10.13)	6.9 (6.30–7.63)	6.6 (4.52–9.51)	6.1 (5.41–6.89)	12.8 (6.28–24.26)	11.8 (9.09–15.25)
	Northeast	26.2 (20.17–33.3)	26.0 (24.07–28.0)	22.7 (16.40–30.56)	22.8 (20.96–24.78)	47.0 (30.46–64.16)	44.7 (37.82–51.75)
	Central-West	7.7 (5.97–9.90)	7.57 (6.76–8.46)	8.0 (6.06–10.58)	7.9 (7.01–8.83)	5.7 (3.49–9.34)	5.8 (4.28–7.79)

MW: minimum wage; 95%CI: 95% confidence interval.

a Minimum wage: 2020 –USD (BRL 1.039,00); 2022 –USD (BRL 1.412,00).

**Figure 1:**
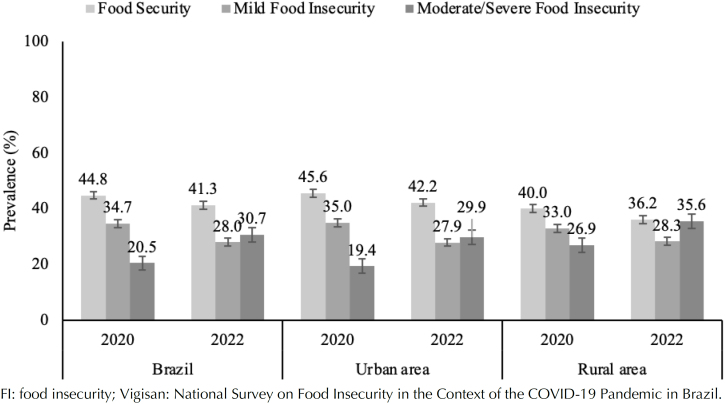
Prevalence of food security and food insecurity levels, Brazil, 2020–2022.

In 2020, more than half of the Brazilian families had FI, and the majority had mild FI (34.7%) or moderate/severe FI (20.5%). The proportion was greater in rural households (mild FI: 28.0%; moderate/severe FI: 30.7%). The prevalence of FI varied from 2020 to 2022, with a notable increase in moderate/severe FI. Although the prevalence of FI in rural areas remained high, one year after the COVID-19 pandemic, the proportion of urban households with moderate/severe FI increased significantly from 19.4% (2020) to approximately 30% (2022) (Figure 1).

**Figure 2:**
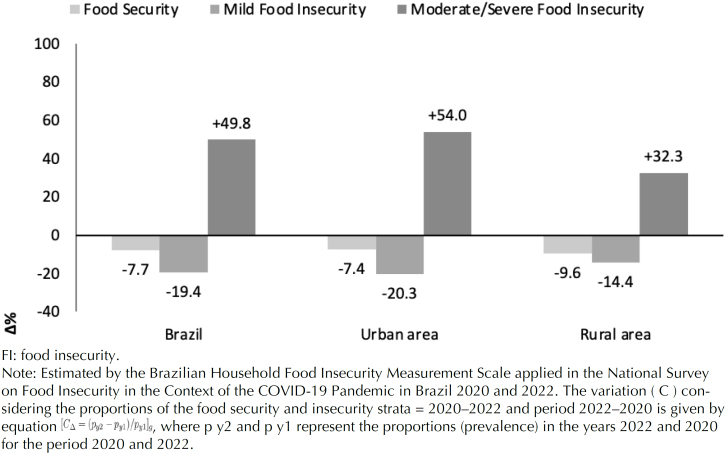
Variation in the prevalence of security, mild and moderate/severe insecurity in rural and urban areas of Brazil. National Survey on Food Insecurity in the Context of the COVID-19 Pandemic in Brazil 2020 and 2022.

Figure 2 shows the variations in the proportions of FS, mild FI and moderate/severe FI for the periods of the two surveys evaluated (Vigisan I = 2020; Vigisan II = 2022). Based on the comparison, a percentage reduction of families experiencing FS in Brazil (-7.7%) was observed, as well as a reduction in mild FI, with a greater reduction among those in the rural areas of the country (-9.6%). However, the percentage variation in moderate/severe FI indicated a significant increase among families across the national territory (+49.8%), with the increase being even greater among families in urban areas in Brazil (+54.0%).

**Figure 3:**
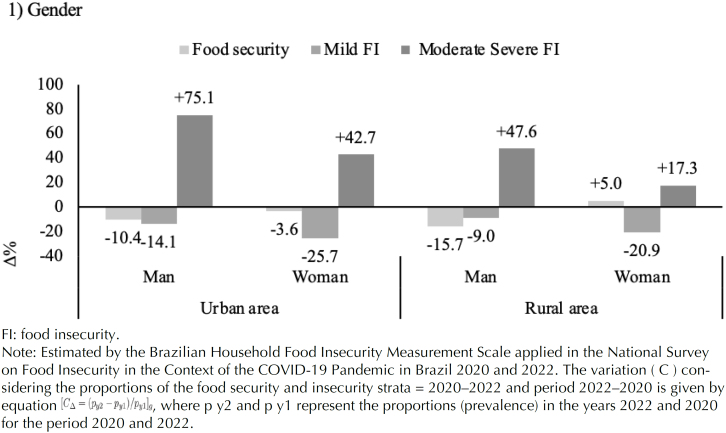
Variation in the prevalence of food security and insecurity according to household heads by gender (man or woman) in urban and rural areas. National Survey on Food Insecurity in the Context of the COVID-19 Pandemic in Brazil 2020 and 2022.

Comparing the variations in FS and FI levels according to the sex of the household heads, urban families headed by men had the lowest variation in the percentage of FS (-10.4) and the greatest variation for the most severe forms of FI (+75.1%). In families in rural areas, this pattern was similar; however, the percentage variation in moderate/severe FI was 2.5 times lower for households headed by women in urban areas (+42.7%) than for those in rural areas (+17.3%) (Figure 3).

**Figure 4:**
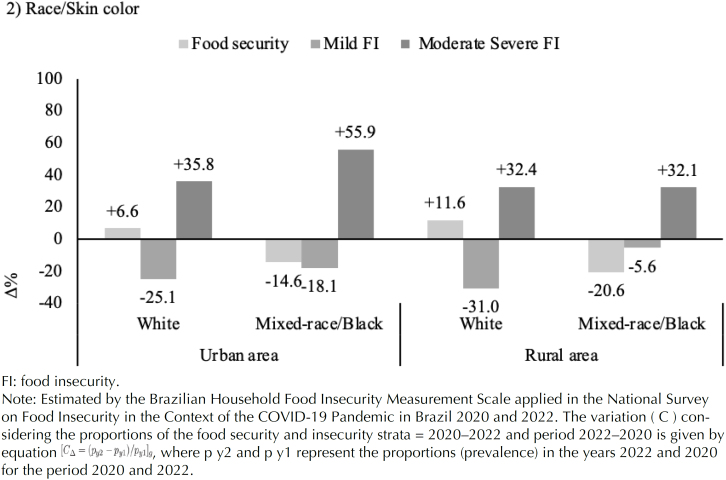
Variation in the prevalence of food security and insecurity according to household heads by people self-declared (white or mixed-race/black) in urban and rural areas. National Survey on Food Insecurity in the Context of the COVID-19 Pandemic in Brazil 2020 and 2022.

Figure 4 shows the variation in FS and FI according to the race/colour of the head of the family. The percentage variation results indicate that only in families headed by a self-declared white person was there an increase in the variation in FS, both in urban areas (+6.6%) and in rural areas (+11.6%). Mild FI decreased more in families headed by a white person from a rural area. The greatest percentage change in the most severe forms of FI was observed among families headed by people self-declared as mixed-race/black from urban areas (+55.9%) compared to those from rural areas (+32.1%).

## DISCUSSION

Considering the period of the COVID-19 pandemic, urban and rural areas presented different socioeconomic profiles, highlighting the higher level of education of heads of households and per capita income in urban areas when compared to rural areas. Between 2020 and 2022, an increase was observed, mainly in the most severe levels, of FI in rural areas. Despite this, there was a greater proportion of households with moderate/severe FI in urban areas.

The percentages of FI observed in urban and rural areas were higher than those published before the pandemic^3,6^. Although there was an increase in FI in 2018, during the pandemic, there was a worsening of moderate/severe FI in rural areas, from 19.3 (2018) to 35.6% (2022), and, in urban areas, from 11.6 (2018) to 29.9% (2022)^8,18^. These percentages reached even lower levels than those observed in the first round of FI monitoring in the country in 2004, a period when food was not seen as a constitutional right and policies aimed at combating hunger and poverty were not yet implemented^6^.

The rural area of Brazil is more vulnerable based on social and demographic indicators and, during the pandemic, was more impacted by poverty^9^. Although income is one of the main factors of FI in the country^19^, this indicator alone was not sufficient to explain the lower proportion of moderate/severe FI in rural areas than in urban areas.

The guarantee of territorial rights is also important for FS. Thus, the stability of land tenure in rural areas can promote food security by guaranteeing the planting, production and sale of food for self-consumption and income generation^9^. On the other hand, during the pandemic, there were barriers to the distribution and commercialization of food, affecting the flow of food production and lowering the sales value of cultivated food, which mainly impacted family farmers^5,8^.

Moreover, in urban centres, the need for social isolation led to an increase in unemployment and informal work, with consequences for household income^20^. The health crisis was also associated with political and economic crises that resulted in a reduction in purchasing power and an increase in the prices of basic foods^5,21^. This situation affected many urban families, causing them to lack money to purchase food, even falling into debt, as they needed to divide their expenses, for example, between housing, food and health^8^.

In Brazil, FI is measured using the EBIA; this makes it possible to compare and understand particular aspects of the housing area and its association with socioeconomic and demographic aspects, even during the COVID-19 pandemic^7,8,10^. Some studies point to gender and race/skin colour inequalities as social determinants of FI^12,22^. This was likewise observed in this study, given that families headed by women and mixed-race/black people were more impacted by severe forms of FI. In the study by Rodrigues et al.^12^, the authors observed that, during the pandemic, the population did not qualitatively share the same experiences of hunger due to social inequalities associated with gender discrimination and racism.

In 2022 in Brazil, six in ten households with female heads experienced some level of FI, and, of these, approximately 20% were in a situation of hunger^8^. During the pandemic, women were socially affected by the increase in working hours and so-called "multitasking", especially those who were mothers and who had to address the closure of schools and the reorganization of the domestic environment^12,22^. Galindo et al.^10^ showed that severe FI in households headed by women was almost double that in households headed by men.

Men have a greater share of household income than women^12^; however, during the pandemic, there was an increase in unemployment and informal work, meaning that men had to accept jobs with lower wages or even informal work to provide income for their dependents. This situation caused men to be more exposed to the disease caused by COVID-19 infection and even resulted in higher male mortality in Brazil^23^.

During some periods of the pandemic, emergency aid, even in insufficient amounts, was essential for the subsistence of many women, who began to depend on the solidarity of neighbours and nonprofit organizations through food donations to meet their food needs^24^. According to Rodrigues et al.^12^, the absence of pensions and social benefits aimed at this population would cause these households to be extremely impacted by FI and social inequalities.

In rural territories, women practice family farming and produce a variety of foods for self-consumption^9^. However, women living in urban areas, especially those living in suburbs and favelas/slums with lower wages, work and education opportunities, faced successive violations of the human right to adequate and healthy food^20,24^, associated with urban violence and difficulties accessing health, education and food.

The pandemic also represented a risk not only for men and women but also for the black population. Although 55.5% of the Brazilian population is composed of mixed race/black people^25^, scholars point out that the most severe levels of FI are associated with this population^10^. During the pandemic, families headed by black people became more vulnerable to severe levels of FI, whereas in 2022 six in ten families with mixed-race/black household heads experienced some level of FI^8,10^.

According to Oxfam Brasil^26^, a Brazilian organisation that is part of a global movement to combat poverty, inequality and injustice, mixed-race/black people were the most affected by unemployment, with a decrease in income from work compared with white people. Notably, black workers constitute the majority of informal wage earners, and unemployed workers were strongly impacted by the pandemic^20,23^.

The results of this study indicate a potential relationship between racial inequality and access to food, especially for household heads who self-declare as mixed-race/black in urban areas. The vast majority of this population are residents of favelas and the city outskirts and suffer from increased fatality due to violence and FI, which reflects the neglect of public authorities and the absence of the state^23,24^.

The present study has several limitations. Budget restrictions and the context at the height of the COVID-19 pandemic did not allow Vigisan I to adopt more robust sampling, such as that conducted in Vigisan II. Even so, as the two surveys used the same sampling plan and the same census sectors adopted in population surveys, it is assumed that the population surveys are comparable to each other and to the surveys conducted by the IBGE^7,8^.

The use of the 8-question version of the EBIA was intended to reduce the interview time and thus reduce the time the interviewers spent with the household heads, limiting their potential exposure to COVID-19 infection. A previous study based on samples from the Brazilian population^15^ demonstrated that the 8-question version and the 14-question version of the EBIA yield similar FI estimates. Additionally, the hiring of the same data collection team and the training of interviewers by the research coordinators, two of whom were involved in the EBIA validation process in Brazil, contributed to reducing bias.

It is worth noting that the COVID-19 pandemic has brought challenges associated with the worsening of social inequalities and access to adequate and healthy food in the country. It is noteworthy that the increase in the most severe forms of FI requires the Federal Government to urgently implement and reformulate public policies and programs aimed at promoting FNS, especially in the post-pandemic period experienced from 2020 onwards.

At the beginning of 2023, the country returned to its anti-hunger agenda, reestablishing the monitoring, planning, and evaluation of FNS policies, along with a series of dialogues on the importance of a critical view of the territory^27^. This article contains data that direct and corroborate the need to structure public policy actions and strategies aimed at eradicating hunger and guaranteeing the human right to adequate food through healthy and sustainable food systems. This structuring will be essential to reduce the social inequalities that increased during the pandemic, especially in urban families.

Therefore, the need to monitor the hunger scenario in the country is reiterated, using the FI indicator applied in population studies, as well as government actions at different levels (federal, state and municipal), especially those aimed at reducing hunger and social inequalities in the Brazilian population.

## CONCLUSION

During the COVID-19 pandemic, inequalities were multiple, complex and interconnected, reproducing various social injustices and violations of human rights, including access to adequate and healthy food in urban and rural areas of Brazil.

The higher proportion of FI in urban areas may be related to unemployment, as well as failures in food supply in urban centres and increased food prices, which mainly impacted male and self-declared mixed-race/black household heads. It is believed that the presence of farmland in rural areas enabled families to produce food for self-consumption, promoting lower impacts of the pandemic in these territories, especially among women.

The results of this study contain important information for analysing the effects of the pandemic on FI in Brazil. The data presented must be examined in light of the reconstruction and strengthening of public social policies, as well as the development of goals and actions adapted to the needs of urban areas and cities. Thus, this article reinforces the need for continued FI monitoring in Brazil, especially considering the current political and social context of the reconstruction of public policies and social programs in the post-pandemic period.

## Data Availability

The datasets generated and/or analyzed during the present study are not publicly available due to [ethical/legal/privacy] restrictions, but are available from the corresponding author upon request.
